# 3D-Printed All-Dielectric Electromagnetic Encoders with Synchronous Reading for Measuring Displacements and Velocities

**DOI:** 10.3390/s20174837

**Published:** 2020-08-27

**Authors:** Cristian Herrojo, Ferran Paredes, Ferran Martín

**Affiliations:** CIMITEC, Department of Electronic Engineering, Universitat Autònoma de Barcelona, 08193 Bellaterra, Barcelona, Spain; ferran.paredes@uab.cat (F.P.); ferran.martin@uab.cat (F.M.)

**Keywords:** microwave sensors, motion control, 3D printing

## Abstract

In this paper, 3D-printed electromagnetic (or microwave) encoders with synchronous reading based on permittivity contrast, and devoted to the measurement of displacements and velocities, are reported for the first time. The considered encoders are based on two chains of linearly shaped apertures made on a 3D-printed high-permittivity dielectric material. One such aperture chain contains the identification (ID) code, whereas the other chain provides the clock signal. Synchronous reading is necessary in order to determine the absolute position if the velocity between the encoder and the sensitive part of the reader is not constant. Such absolute position can be determined as long as the whole encoder is encoded with the so-called de Bruijn sequence. For encoder reading, a splitter/combiner structure with each branch loaded with a series gap and a slot resonator (each one tuned to a different frequency) is considered. Such a structure is able to detect the presence of the apertures when the encoder is displaced, at short distance, over the slots. Thus, by injecting two harmonic signals, conveniently tuned, at the input port of the splitter/combiner structure, two amplitude modulated (AM) signals are generated by tag motion at the output port of the sensitive part of the reader. One of the AM envelope functions provides the absolute position, whereas the other one provides the clock signal and the velocity of the encoder. These synchronous 3D-printed all-dielectric encoders based on permittivity contrast are a good alternative to microwave encoders based on metallic inclusions in those applications where low cost as well as major robustness against mechanical wearing and aging effects are the main concerns.

## 1. Introduction

Optical encoders are widely used in many applications such as the automotive industry, elevators, robotics, and conveyor belts, among others, in order to accurately measure linear or angular displacements and velocities [1,2,3]. Usually, such optical encoders are based on a chain (or more generally, a grid) of apertures in a metallic plate (a disc in rotary encoders), which moves with regard to the reader, essentially constituted by a light source and an optical detector. The displacement and/or velocity information of the encoder is determined from the optical pulses received in the detector, as the chain of apertures of the metallic plate is displaced (linearly or angularly) across the optical path between the source and the detector. Typically, optical encoders exhibit very good position resolution. Nevertheless, the reliability of optical encoders is reduced in harsh environments, e.g., with pollution, grease, dirtiness, etc.

Electromagnetic (or microwave) encoders can be a good alternative to optical encoders, since microwaves are more tolerant to the effects of pollution than optical signals. Moreover, microwave encoders represent a low-cost solution in motion control applications. In recent years, many different microwave sensors for measuring angular or linear displacements and velocities have been reported. In [4,5,6,7,8,9,10], the microwave sensors are based on a transmission line loaded with a resonant element, which is etched in a different substrate and positioned in close proximity to the transmission line. Typically, the rotation angle or linear displacement of the resonant element with regard to the transmission line is determined from a change in the frequency response of the whole structure (bandwidth, quality factor, resonance frequency, etc.). The main drawback of these approaches is the limited dynamic range. In [11,12,13,14,15], microwave encoders based on a linear or angular chain of metallic inclusions implemented on a dielectric substrate were reported. For encoder reading, a passive structure fed by a harmonic signal, able to detect the presence or absence of such inclusions (located at predefined and equidistant positions on the considered substrate), was designed. The main drawback of this approach is the limited robustness against mechanical wearing (e.g., due to friction) and aging effects. To alleviate this problem, all-dielectric microwave encoders based on permittivity contrast were reported in [16,17]. In such encoders, the metallic inclusions were replaced with apertures or dielectric inclusions made on the considered substrate, and the sensitive part of the reader was designed in order to detect the presence/absence of apertures (or dielectric inclusions). In order to reduce the cost of these all-dielectric encoders, the functionality of 3D-printed microwave encoders was demonstrated in [17]. It is important to mention that, in both types of microwave encoder (either based on metallic inclusions or based on apertures), the position and velocity of the encoder were extracted by considering a complete chain of inclusions (i.e., with all the inclusions present at their predefined positions). The position is determined by counting pulses, which are generated at the output port of the sensitive part of the reader by encoder motion, whereas the velocity is inferred from the time interval between adjacent pulses (provided that the period of the inclusions’ chain is well known). Nevertheless, note that, with this approach (regardless of the considered inclusions), it is not possible to determine the absolute position of the encoder, since such a position is determined from the cumulative number of pulses. Therefore, if the system suffers a reset, the encoder should be driven to a reference position.

To solve the previously cited limitation, a new approach for microwave encoders was reported in [18,19,20,21]. Specifically, the encoders are based on two metallic inclusion chains. One chain contains a certain identification (ID) code (where the presence and absence of an inclusion in a specific position of the encoder is interpreted as a “1” or “0” logic state, respectively), and another one (with all metallic inclusions present) is used as a clock, in order to properly read the position chain. Moreover, the clock chain provides the instantaneous velocity (or even the acceleration) of the encoder. It is important to highlight that this approach can also be applied to chipless RFID systems based on near-field coupling and sequential bit reading, in order to synchronously read the ID code if the relative velocity of the encoder with regard to the sensitive part of the reader is not constant [22,23,24,25,26,27,28,29,30,31,32].

This paper contributes to the state of the art of electromagnetic encoders with synchronous reading (as compared to encoders based on metallic inclusions) in two main aspects: (i) reduced cost due to the fact that the proposed encoders of this work are fabricated with low-cost 3D printing materials and (ii) very high robustness against exposure to harsh environments. For instance, the encoders do not deteriorate when they are in contact with liquid or water. It is important to mention that, to this end, a specific reader has been designed and fabricated.

The paper is organized as follows. The working principle, as well as the design of the low-cost 3D-printed all-dielectric encoders based on permittivity contrast with synchronous reading, is presented in Section 2. Experimental validation is reported in Section 3, where the functionality of the encoders as displacement/velocity sensors is demonstrated. Finally, the main conclusions are highlighted in Section 4.

## 2. Reader and Encoder Design

The considered encoders are based on dielectric inclusions, dedicating one part to the ID code and the other part to the clock signal. This is indeed equivalent to considering a pair of parallel inclusion chains, one devoted to the ID code and the other one to the clock signal (the strategy adopted in [21]). Naturally, in the ID chain, encoding is achieved by the presence (“1” logic state) or absence (“0” logic) state of functional inclusions. By contrast, all the inclusions are present (and they are functional) in the clock chain.

In this paper, chains of linear apertures in a 3D-printed material, orthogonally oriented to the chain axis and closely spaced, constitute an optimum strategy for resolution and data density optimization. These encoders were first reported in a recent paper by the authors (without synchronous reading capability) [17]. In [17], the sensitive part of the reader was a microstrip line loaded with a series gap and a transversally oriented slot resonator etched in the ground plane, beneath the gap. It was demonstrated in [33,34] that the resonance frequency of slot resonators is very sensitive to the dielectric constant of the surrounding medium. This explains the use of a linear slot resonator (with dimensions similar to those of the linear apertures of the encoder) as the key element for reader implementation. The series gap merely introduces a pole in the frequency response, convenient to achieve a significant excursion of the transmission coefficient at the operating frequency, *f_c_*, i.e., the frequency of the interrogation signal. Such frequency was tuned to the pole frequency of the bare slot resonator in [17], which roughly coincides with the resonance frequency of the resonator covered with the considered encoder substrate [17].

In [17], a single harmonic interrogation signal was needed in order to detect the presence of the apertures (and thus determine the ID code) by encoder motion. In brief, the working principle is as follows: an amplitude modulated (AM) signal is generated at the output port of the reader line as a consequence of encoder motion, by virtue of the different transmission coefficient at *f_c_* that results when an aperture is perfectly aligned with the slot resonator. Hence, the envelope function of this AM signal exhibits peaks corresponding to the encoder apertures [17], and the ID code can be inferred by means of an envelope detector. However, since the encoder of this work contains two independent chains, further system complexity is needed. Particularly, two slot resonators tuned to different frequencies must be considered for synchronous encoder reading. One such resonator must be placed beneath the ID code chain and the other beneath the clock chain. Obviously, two independent harmonic signals are also necessary in order to obtain the clock signal and the ID code.

**Figure 1:**
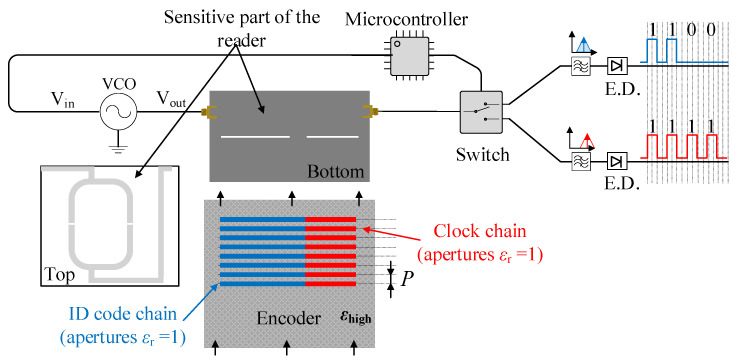
Sketch showing the working principle of the proposed reader/encoder system based on permittivity contrast with synchronous reading.

A sketch of the conceived system is depicted in Figure 1, where a splitter/combiner configuration with each branch loaded with a series gap and a transverse slot resonator is considered. According to this sketch, a microcontroller ensures that a voltage-controlled oscillator (VCO) alternately injects both interrogation signals to the input port of the splitter/combiner. Then, in order to separately obtain the clock signal and the ID code, a switch as well as an envelope detector (one for each signal) are added to the output port of the splitter/combiner, as can be appreciated in Figure 1. Since the clock chain apertures and the ID chain apertures (if present) are located at the same axial position, it follows that the clock signal provides the time instants necessary to read the ID code from the corresponding envelope function.

**Figure 2:**
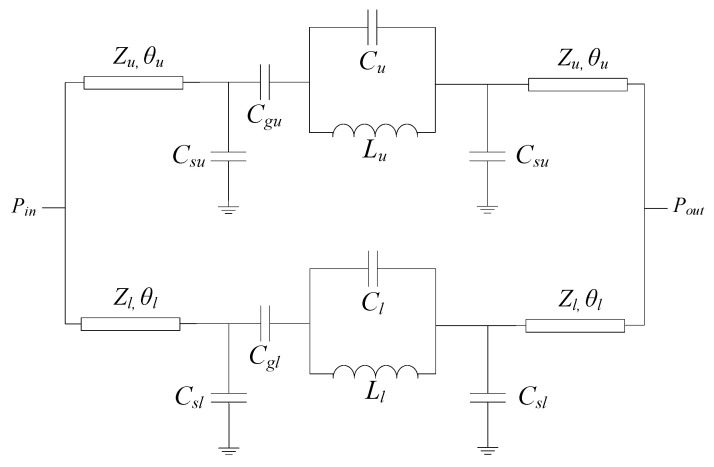
Circuit model of the considered splitter/combiner structure.

The design of the splitter/combiner is important since its relevant dimensions must be adequately chosen for correct system functionality. Concerning the slot resonators, the one in the upper branch has been designed so that it resonates at *f*_0*u*_ = 4.229 GHz, whereas the fundamental resonance of the slot resonator of the lower branch is *f*_0*l*_ = 3.155 GHz. The gap apertures are identical in both branches, and such dimensions have been tuned in order to obtain the poles of the corresponding (isolated) branches close to the resonance frequencies of the slots. The width of the lines in both branches corresponds to a characteristic impedance of *Z_u_* = *Z_l_* = 50 Ω (where the sub-indexes identify the upper and lower branch). Finally, the lengths of the transmission line sections at both sides of the series gaps have been selected so that the open circuit at the resonance frequency of the slot resonators is transformed to a short-to-ground at the Y junction at the corresponding frequencies. This is achieved if the electrical length of the transmission line sections is close to 90° or an odd multiple of such electrical length. In order to determine the specific value of the electrical length for each branch, the circuit model of the splitter/combiner is needed. It is depicted in Figure 2, where *C_u_* (*C_l_*) and *L_u_* (*L_l_*) account for the capacitance and inductance, respectively, of the upper (lower) slot resonator. The upper (lower) series gap is accounted for by means of the series, *C_gu_* (*C_gl_*), and shunt, *C_su_* (*C_sl_*), capacitances [17].

The condition to translate the open circuit at the corresponding slot resonance frequency of a branch to the Y junction of the splitter/combiner is to force that *Y*_11_ = ∞ for this branch (*Y*_11_ being the first element of the admittance matrix). This ensures a transmission zero at the resonance frequency of each slot resonator (i.e., two transmission zeros), regardless of the loading of the other slot resonator. For simplification purposes, let us designate by *Y_Ru_* (*Y_Rl_*) and *Y_Su_* (*Y_Sl_*) the admittances of the series and shunt branches, respectively, of the Π circuits cascaded between the transmission line sections of the upper (lower) branch. From ABCD matrix to Y matrix conversion, it is found that *Y*_11_ = *D*/*B* [35]. Thus, for each splitter/combiner branch, the ABCD matrix is first obtained. For the upper branch, the *B* and *D* elements of this matrix are found to be

(1) $$B_{u}=\frac{{\left({\cos\;\theta }_{u}\right)}^{2}}{Y_{Ru}}-Z_{u}^{2}{\left({\sin\;\theta }_{u}\right)}^{2}\left(2Y_{Su}+\frac{Y_{Su}^{2}}{Y_{Ru}}\right)+2j\left(1+\frac{Y_{Su}}{Y_{Ru}}\right)Z_{u}{\sin\;\theta }_{u}{\cos\;\theta }_{u}$$

(2) $$D_{u}=\left(1+\frac{Y_{Su}}{Y_{Ru}}\right)\left\{{\left({\cos\;\theta }_{u}\right)}^{2}-{\left({\sin\;\theta }_{u}\right)}^{2}\right\}+j\frac{Y_{u}{\sin\;\theta }_{u}{\cos\;\theta }_{u}}{Y_{Ru}}+j\left(2Y_{Su}+\frac{Y_{Su}^{2}}{Y_{Ru}}\right)Z_{u}{\sin\;\theta }_{u}{\cos\;\theta }_{u}$$

with *Y_u_* = 1/*Z_u_*. Note that, although the characteristic impedances of the transmission line sections of both branches are identical (i.e., *Z_u_* = *Z_l_* = 50 Ω, as indicated above), the analysis is carried out by relaxing this requirement, namely by considering arbitrary impedances. In Equations (1) and (2), *θ_u_* is the electrical length of the line sections at both sides of the series gap for the upper branch. Similar expressions to Equations (1) and (2) are obtained for the lower branch by simply replacing the sub-index *u* with the sub-index *l*. Using Equations (1) and (2), *Y*_11,*u*_ (*Y*_11,*l*_) can be evaluated as indicated above. In the limit when *ω* → *ω*_0*u*_ (*ω* → *ω*_0*l*_), with *ω*_0*u*_ = 2π*f*_0*u*_ (and *ω*_0*l*_ = 2π*f*_0*l*_), the admittance of the series branch of the lumped element circuit is an open circuit, i.e., *Y_Ru_* → 0 (*Y_Rl_* → 0), and the first diagonal element of the admittance matrix can be expressed as

(3) $${Y_{11,u}|}_{\omega_{0,u}}=\;\frac{Y_{u}{\sin\;\theta }_{u}{\cos\;\theta }_{u}+j_{\omega_{0u}}C_{su}\left\{{\left({\cos\;\theta }_{u}\right)}^{2}-{\left({\sin\;\theta }_{u}\right)}^{2}-\omega_{0u}C_{su}Z_{u}{\sin\;\theta }_{u}{\cos\;\theta }_{u}\right\}}{{\left({\cos\;\theta }_{u}-\omega_{0u}C_{su}Z_{u}{\sin\;\theta }_{u}\right)}^{2}}$$

where *Y_Su_* = *j ω C_su_* has been used. An equivalent expression results for the lower branch. According to (3), *Y*_11,*u*_ = ∞ if the denominator of Equation (3) is null, that is

(4) $${\cot\;\theta }_{u}=\omega_{0u}C_{su}Z_{u}$$

and, obviously, *Y*_11,*l*_ = ∞ if the electrical length of the transmission line sections of the lower branch satisfies

(5) $${\cot\;\theta }_{l}=\omega_{0l}C_{sl}Z_{l}$$

Inspection of Equations (4) and (5) indicates that, if the fringing capacitance of the series gaps, *C_su_* (*C_sl_*) is null, the electrical lengths must satisfy *θ_u_*(*θ_l_*) = (2*n* + 1) π/2, where *n* = 0, 1, 2, 3,..., as expected. Thus, the effect of the fringing capacitance (inevitable, in practice, but small) is a slight modification, as compared to the ideal case (90° or an odd multiple) of the electrical length necessary to translate the open circuit (present at the central position) to a short circuit at the Y junction. By choosing the electrical lengths according to Equations (4) and (5), there are two transmission zeros in the bare structure which are not consequences of an interfering phenomenon but are determined by the dimensions of the bare slot resonators. By this means, it is guaranteed that both chains of apertures modify only the corresponding transmission zero (i.e., the resonance frequency of the slot aligned with the corresponding chain) when the encoder is in motion, but they do not have any effect on the other transmission zero. This condition is necessary for system functionality.

The specific electrical lengths for the upper and lower transmission line sections have been set to *θ_u_* = 87.7° and *θ_l_* = 88.3°, respectively, as these values are coherent with Equations (4) and (5). To evaluate Equations (4) and (5), we have first inferred the fringing capacitances through the parameter extraction procedure described in [36] and already used in [17]. The extracted element parameters are *C_su_* = *C_sl_* = 30.2 fF. The resonance frequency of each slot resonator is directly given by the first transmission zero of the isolated branch.

**Figure 3:**
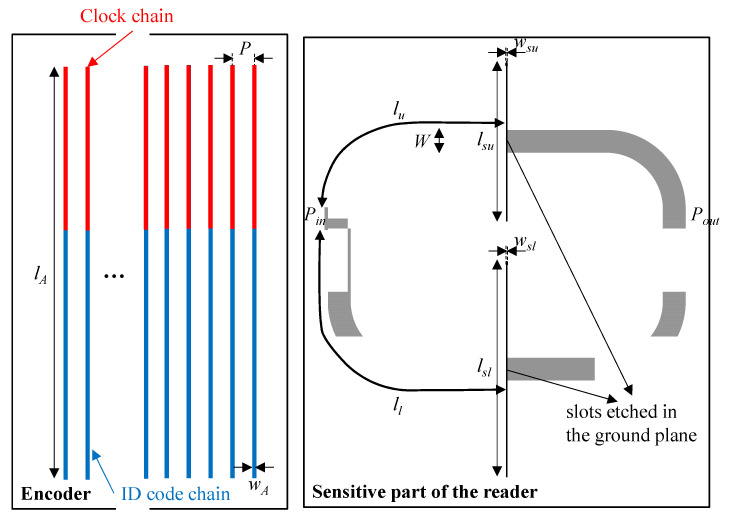
Layout of the designed splitter/combiner structure loaded with a pair of slots and series gaps. Dimensions (in mm) are *W* = 3.40, *l_su_* = 24.1, *w_su_* = 0.20, *l_sl_* = 32.5, *w_sl_* = 0.20, *l_u_* = 33.6, *l_l_* = 44.7, *l_A_* = 62.6, *w_A_* = 0.40, and *p* = 3.40.

**Figure 4:**
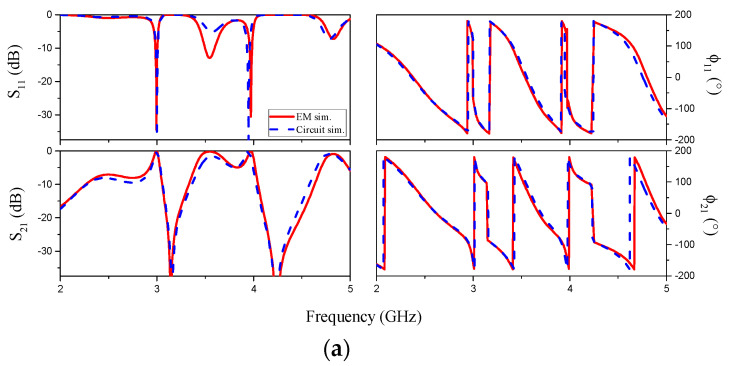

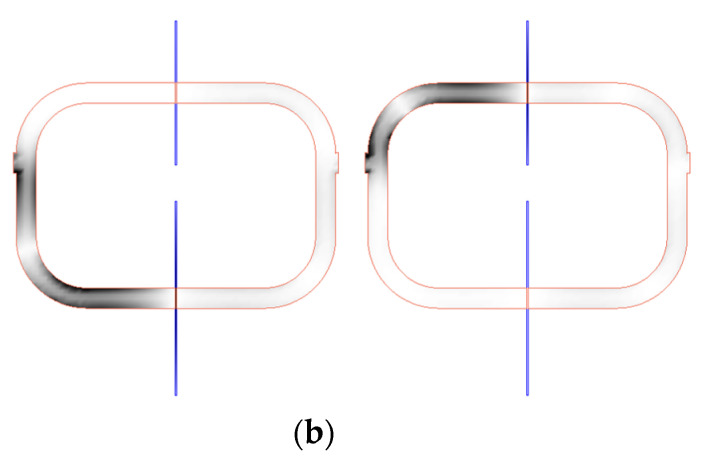
(**a**) Frequency response of the sensitive part of the bare reader inferred from lossless electromagnetic simulation and circuit simulation of Figure 2. The extracted element parameters are *C_su_* = 30.2 fF, *C_gu_* = 0.06 pF, *C_u_* = 0.40 pF, *L_u_* = 3.58 nH, *C_sl_* = 30.2 fF, *C_gl_* = 0.13 pF, *C_l_* = 1.26 pF, and *L_l_* = 2.02 nH. Such simulations have been inferred by means of the Keysight ADS software; (**b**) Current distribution of the sensitive part of the reader at the fundamental resonance frequency of the slot resonator of the upper and lower branch.

**Figure 5:**
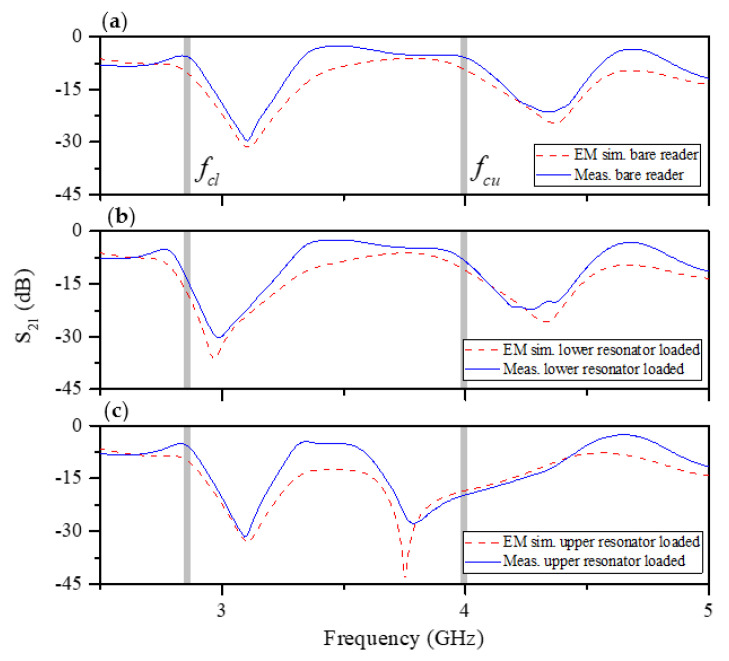
Electromagnetic simulation (including losses) and measured frequency responses of the (**a**) bare reader, (**b**) reader with the longer slot loaded with the RS MT-Copper on top of it, and (**c**) reader with the shorter slot loaded with RS MT-Copper on top of it. The simulation has been inferred by means of the Keysight ADS software

The layout of the designed splitter/combiner structure and relevant dimensions are depicted in Figure 3. It is important to mention that half-wavelength transmission lines have been added to the upper and lower transmission line sections (*θ_u_* and *θ_l_*) in order to be able to implement the splitter/combiner (for electromagnetic and circuit simulation comparison, such half-wavelength transmission line sections have been also added to the circuit model presented in Figure 2). The lossless frequency response of this structure, including the circuit and electromagnetic simulation, is depicted in Figure 4a. Such simulations have been inferred by means of the Keysight ADS software package (Keysight, Santa Rosa, CA, USA), which includes a circuit simulator and an electromagnetic solver (Keysight Momentum). The agreement is very good, thereby pointing out the validity of the model. Moreover, in Figure 4b, it is depicted the current distribution of the sensitive part of the reader at the fundamental resonance of the slot resonator of the upper and lower branch, *f*_0*l*_ = 3.155 GHz and *f*_0*u*_ = 4.229 GHz, in order to demonstrate that, at these frequencies, the current is maximal at the input port (input impedance is zero). The structure has been fabricated by means of the LPKF H100 drilling machine (LPKF Laser & Electronics, Garbsen, Germany) in the Rogers RO4003C substrate (Rogers Corporation, Chandler, AZ, USA) with dielectric constant *ε_r_* = 3.55, thickness *h* = 1.52 mm, and loss tangent tan*δ* = 0.0021. The measured response of the sensitive part of the reader has been obtained by means of the Agilent 5221A vector network analyzer (Keysight, Santa Rosa, CA, U.S.A.) and compared with the electromagnetic simulation (including losses). The results, which are depicted in Figure 5, indicate that, by loading only one of the slot resonators by means of the considered dielectric material (the one of the encoders), the resonance frequency shifts, with the result of a modification in the corresponding transmission zero, but leaving unaltered the other transmission zero. This can be appreciated by inspection of the measured responses inferred by alternately loading the slot resonators. Such dielectric material is the RS Pro MT-Copper. In [17], the measured dielectric constant and loss tangent of this material (inferred by means of the resonant cavity Agilent 85072A, (Keysight, Santa Rosa, CA, USA) were found to be *ε*_r_ = 7.6 and tan*δ* = 0.015, respectively. As will be shown next, the permittivity contrast between the considered material (host substrate) and air (apertures) is enough to achieve significant variation of the transmission coefficient at the interrogation signals (*f_cl_* and *f_cu_*).

## 3. Experimental Validation

**Figure 6:**
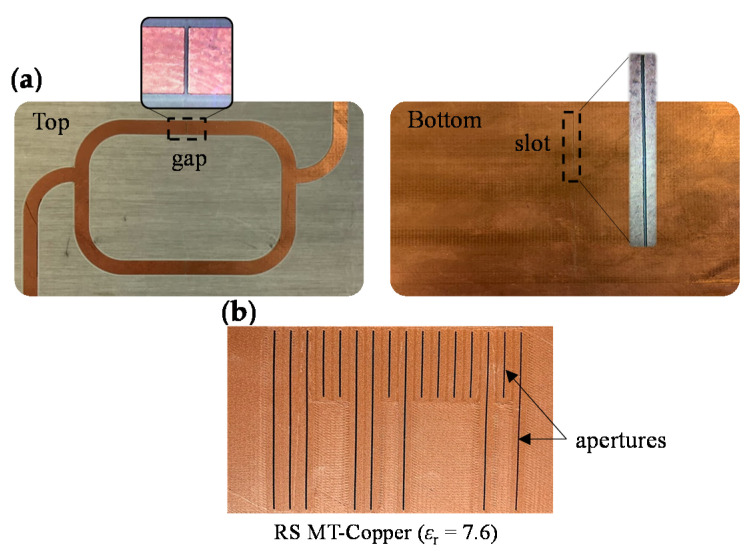
Photographs of the fabricated (**a**) sensitive part of the reader and (**b**) 3D-printed 16-bit encoder codified with a de Bruijn sequence.

We have fabricated a 16-bit encoder by means of the Ultimaker 3 Extended 3D printer (Ultimaker, Utrecht, The Netherlands) with the dielectric material indicated before. This 3D printer provides enough resolution for our purposes. Concretely, the maximum resolution of this 3D printer in the *x*, *y*, and *z* directions is 12.5 μm, 12.5 μm, and 2.5 μm, respectively [37]. On the other hand, the sensitive part of the reader has been implemented by means of the milling machine LPKF-100 (LPKF Laser & Electronics, Garbsen, Germany) on the substrate RO4003C (with dimensions, dielectric constant, and loss tangent also mentioned before). The photograph of the 16-bit encoder and the sensitive part of the reader are depicted in Figure 6. The encoder has been codified with a de Bruijn sequence, which guarantees that, for a given sequence of length *k*, any *n*-bit sub-code appears only once [38]. Thus, the absolute position of the encoder can be univocally identified by reading a bit of the ID code chain and the previous *n*-1 bits (in our case, *n* = 4).

**Figure 7:**
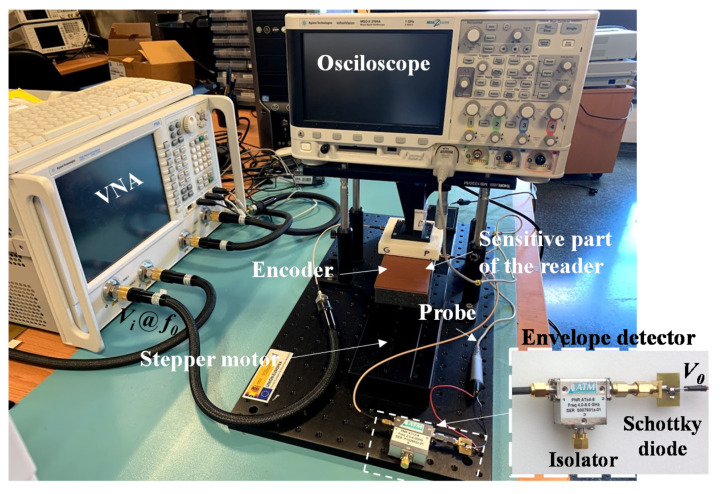
Photograph of the experimental setup.

**Figure 8:**
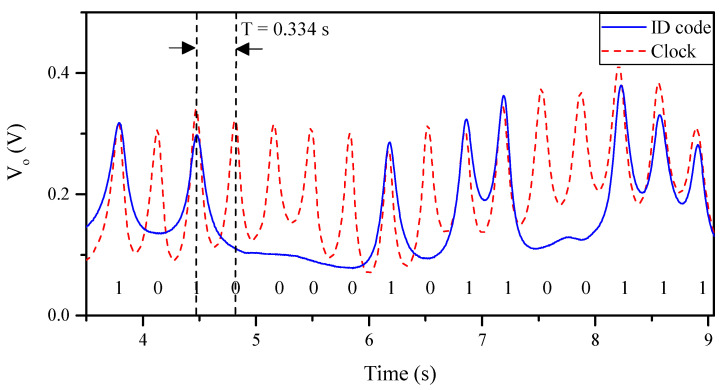
Measured envelope functions (ID code and clock signals) of the 16-bit encoder with the indicated code.

The photograph of the experimental set-up is shown in Figure 7. As aforementioned, it is necessary to generate two interrogation signals (tuned to *f_cu_* and *f_cl_*) to synchronously read the ID code of the encoder. It is important to mention that, as a proof of concept demonstrator, such interrogation signals have been injected independently to the sensitive part of the reader by means of a vector network analyzer (model Agilent N5221A, Keysight, Santa Rosa, CA, USA). For visualizing the ID code of the encoder, an oscilloscope (model Agilent MSO-X-3104A, Keysight, Santa Rosa, CA, USA) has been used. The envelope function of the AM modulated signal generated by tag motion at the output port of the sensitive part of the reader has been extracted by means of an envelope detector, which is comprised of a Schottky diode (model Avago HSMS- 2860, Broadcom Limited, San Jose, CA, USA) and the N2795A active probe (with capacitance *C* = 1 pF and resistance *R* = 1 MΩ, Keysight, Santa Rosa, CA, USA). Moreover, it should be noted that a circulator (model ATM ATc4-8, L3Harris Narda-MITEQ, Hauppauge, NY, USA), acting as isolator, has been added between the sensitive part of the reader and the envelope detector in order to avoid reflections from the Schottky diode. Finally, the relative displacement between the sensitive part of the reader and the encoder has been achieved by means of a linear displacement system (model STM 23Q-3AN, Applied Motion Products Inc., Watsonville, CA, USA). Such a system allows us to control the vertical distance (air gap) between the sensitive part of the reader and the encoder, the velocity, and even the acceleration of the encoder. Considering this setup for experimental validation, the measured clock signal and ID code of the fabricated 16-bit encoder has been inferred, as depicted in Figure 8. Such measured responses have been obtained by displacing the encoder over the sensitive part of the reader with a constant velocity of 10 mm/s. Note that the clock signal perfectly determines the time intervals for synchronously reading the de Bruijn sequence in the encoder chain (revealed as peaks in the envelope function). Hence, the absolute position of the encoder can be determined. Moreover, the measured relative velocity between the encoder and the sensitive part of the reader has been found to be 10.17 mm/s (from the time interval between adjacent peaks of the clock signal), which is in good agreement with the nominal value (10 mm/s). Note that the displacement direction cannot be detected with this approach. Nevertheless, such information might be extracted (by software) from the linear displacement stepper motor used in the experimental set-up. 

**Figure 9:**
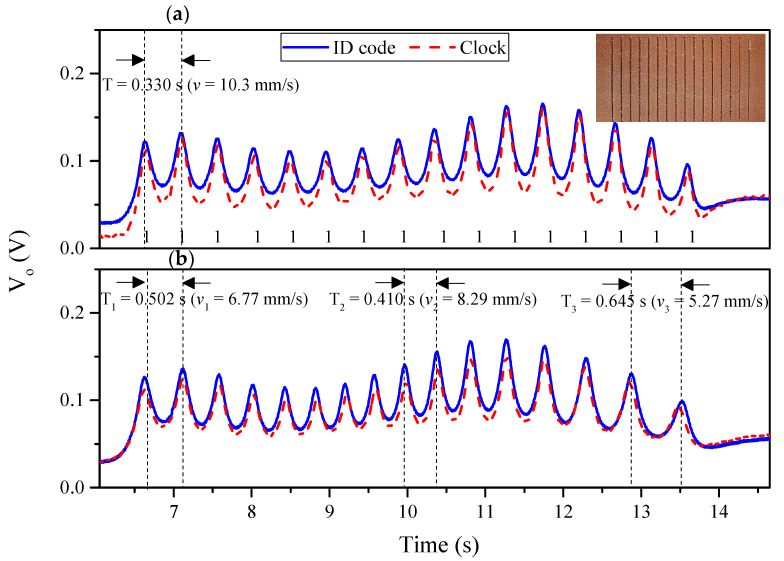
Photograph and measured envelope functions (ID code and clock signals) of the 3D-printed 16-bit encoder with all the apertures present at their positions, inferred by considering encoder motion with (**a**) constant velocity and with (**b**) constant acceleration.

As indicated in the introduction, the encoder position, or displacement, from a well-known reference position can be determined from the cumulative number of pulses (peaks in the envelope function) of the clock signal, provided that the chain period (*p*) is well known. For validation purposes, we have fabricated a 3D-printed 16-bit encoder with all the apertures present in the ID code chain. The photograph and measured envelope function (ID code and clock signal) of the fabricated 16-bit encoder, by considering (i) constant velocity and (ii) constant acceleration of the encoder with regard to the sensitive part of the reader, are depicted in Figure 9. With this result, it is demonstrated that, by counting pulses, it is possible to infer the position of the encoder. Moreover, the ID code can be properly obtained regardless of the instantaneous velocity of the encoder. Nevertheless, this approach presents two main drawbacks: (i) it is not possible to determine the absolute position of the encoder, and (ii) a reference position is needed in order to drive the encoder to such position if the system is restarted for any reason. Conversely, by encoding the ID code chain of the encoder with a de Bruijn sequence, only *n* bits of such ID code chain must be synchronous read after a system reset, in order to determine again the absolute position of the encoder.

The number of bits (or pulses) per unit length (a figure of merit) achieved with the proposed reader/encoder structure with synchronous reading is as high as 3.11 bit/cm. As compared to other linear displacement/velocity sensors based on microwave encoders with synchronous reading, such value is very competitive. For instance, in [21], the achieved number of bits per unit length was 2.29 bit/cm (to the best of our knowledge, the highest value obtained so far). Nevertheless, the microwave encoders of [21] are based on metallic inclusions implemented on the considered substrate. Such encoders exhibit limited robustness against mechanical wearing and aging effects, as compared to all-dielectric microwave encoders based on permittivity contrast. Moreover, as demonstrated in [17], the cost of the considered 3D-printed materials is smaller than the one corresponding to commercial microwave substrates or even to that of microwave encoders based on printed metallic inclusions (where commercial conductive inks are used).

An important aspect in these encoder systems is the effect of the variation in the air gap (vertical distance between the encoder and the sensitive part of the reader), which can be caused by vibrations in the system. This aspect was studied in [17], which revealed that, by considering an air gap of 0.075 mm, it was possible to obtain a significant variation in the magnitude of the transmission coefficient at the carrier frequency signal. Such variation is reduced as the air gap increases. Concretely, for air gaps greater than 0.1 mm, the reader is almost insensitive to the presence of the encoder. Therefore, in a real scenario, the guiding system must ensure a vertical distance of the order of 0.1 mm between the encoder and the sensitive part of the reader.

## 4. Conclusions

All-dielectric 3D-printed electromagnetic encoders based on permittivity contrast with synchronous reading, useful for measuring displacements and velocities, have been reported for the first time. The encoders are based on linearly shaped apertures made on a 3D-printed material which exhibits high permittivity. The sensitive part of the reader is a splitter/combiner structure, where each branch has been loaded with a series gap and a slot resonator. Such a structure is able to detect the presence of the apertures, provided that they are at a short distance. Thus, by feeding the sensitive part of the reader with two interrogation signals properly tuned, two AM modulated signals are alternately generated at the output port of the structure by encoder motion. The envelope function of one such signal contains the ID code providing the absolute position of the encoder, whereas the velocity, or even the acceleration, is given by the other envelope function (acting as a clock signal for synchronous reading). The number of bits per unit length has been significantly increased as compared with other microwave encoders available in the literature. Moreover, the reported all-dielectric encoders exhibit the following advantages as compared to microwave encoders based on metallic inclusions: (i) reduced cost and (ii) major robustness against mechanical wearing and aging effects.
